# Characterization of a novel antibiofilm effect of nitric oxide-releasing aspirin (NCX-4040) on *Candida albicans* isolates from denture stomatitis patients

**DOI:** 10.1371/journal.pone.0176755

**Published:** 2017-05-11

**Authors:** Francisco Madariaga-Venegas, Roberto Fernández-Soto, Luisa Fernanda Duarte, Nicole Suarez, Daniela Delgadillo, José A. Jara, Ricardo Fernández-Ramires, Blanca Urzúa, Alfredo Molina-Berríos

**Affiliations:** 1 Institute for Research in Dental Sciences, Faculty of Dentistry, University of Chile, Santiago, Chile; 2 Department of Pathology and Oral Medicine, Faculty of Dentistry, University of Chile, Santiago, Chile; Virginia Commonwealth University, UNITED STATES

## Abstract

*Candida albicans* biofilms play a key role in denture stomatitis, one of the most common oral pathologies in elderly people. Because biofilms are highly resistant to antifungals, new pharmacological strategies are needed. Aspirin and nitric oxide-donor molecules have both shown antibiofilm effects on *C*. *albicans*, making them promising candidates for treatment. In this study, we evaluated the antifungal/antibiofilm effect of a nitric-oxide releasing aspirin (NO-ASA) on *C*. *albicans* isolates from denture stomatitis patients *in vitro*. Disk diffusion assays showed that while NO-ASA had no antifungal effect, the drug potentiated fluconazole inhibition zone diameters, increasing the effect of fluconazole by 20–30% (p<0.05). The effect of NO-ASA on the morphogenesis of *C*. *albicans* was evaluated using light microscopy after inducing hyphae formation. For all clinical strains assayed, 125 μM NO-ASA significantly decreased the number of filamentous cells present (p<0.01). Adhesion to abiotic surfaces, a critical event for biofilm formation, was evaluated in 96-well polystyrene plates using crystal violet assay; 125 μM NO-ASA significantly inhibited adhesion. Biofilms were observed with scanning electron microscopy (SEM) and quantified using XTT reduction assay. NO-ASA decreased biofilm formation (IC_50_ ranging from 300 μM to 700 μM), consistent with SEM findings of altered biofilm microarchitecture. PGE_2_ and carboxy-PTIO (an NO scavenger) both blocked the antibiofilm effects of NO-ASA, suggesting that the efficacy of NO-ASA may be associated with both inhibition of PGE_2_ synthesis and release of NO. NO-ASA is a promising novel antibiofilm agent for treating fluconazole-resistant strains of *C*. *albicans*.

## Introduction

Denture stomatitis (DS) is one of the most common oral diseases in the elderly. DS is often related to an oral *Candida albicans* infection. The condition is characterized by inflammation of the mucosa beneath the denture, where *Candida* spp. biofilms contribute significantly to perpetuation of the disease and resistance to antifungal treatment [1]. Clinically, DS induces symptoms ranging from mild, localized inflammation to inflammatory papillary hyperplasia, and in susceptible patients, DS is associated with numerous other complications [2]. The etiology of this pathology is multifactorial, but it is generally associated with trauma or infection. Risk factors include systemic disease, immunosuppression, decreased salivation, continuous use of denture prostheses, use of certain drugs (especially antibiotics and corticosteroids), cigarette smoking, and poor oral hygiene [2]. Although access to dental care has improved in recent decades, the prevalence of DS remains as high as 60–70%, even in developed countries [3].

*Candida* spp. form biofilms on diverse biomaterials and are frequently present on denture prostheses. *Candida albicans* is the most prevalent species of the genus [2,4] and is part of the normal oral cavity flora, where it rarely causes disease. However, in immunocompromised patients, *Candida albicans* can provoke complications ranging from superficial infections to invasive systemic candidiasis [5]. The ability of *C*. *albicans* to form biofilms increases its resistance to treatment, as antifungals are often ineffective against established biofilms [6]. In a recent literature review, Yarborough and colleagues reported that conventional antifungal treatments often lead to positive clinical responses at least in the short term; however, as there were was little data available to support the long-term efficacy of these drugs as most of the studies were short in duration [7]. Other studies indicate that antifungal therapies not only have limited long-term efficacy but are also associated with long-term emergence of drug-resistant strains of *C*. *albicans* and severe stomatitis [8].

*C*. *albicans* biofilms consist of yeast-form cells and long, tubular hyphal cells, both of which play unique roles in biofilm formation [9]. The first step of biofilm formation is adhesion of planktonic yeast cells to the biomaterial or host cells, a process mediated by nonspecific interactions and activation of specific proteins called adhesins [5]. The yeast cells then proliferate across the surface of the host, generating projections that grow into filamentous forms (hyphae and pseudohyphae). Simultaneously, the cells produce an abundant protective extracellular matrix that accumulates as the biofilm matures, contributing to antifungal drug resistance [10,11]. In the final stage of the biofilm cycle, yeast is released from the biofilm to the surrounding medium to form additional biofilms or disseminate and colonize new tissues [9]. While it is clear that biofilms are highly resistant to antifungal drugs, the underlying mechanisms are not fully understood. It is possible that this resistance is the result of a series of processes rather than a single element.

*C*. *albicans* biofilms are highly resistant to azoles, including the newest drugs in this class, voriconazole and posaconazole. Cells living in biofilms are up to 1000-fold more resistant to fluconazole than planktonic cells, and triazoles are 50% less effective on *C*. *albicans* that inhabit biofilms as compared to their free-living counterparts [12]. Although topical drugs are the most common strategy for controlling lesion progression [13], patients with immunosuppression or other special needs often require systemic antifungals [13]. Several studies have reported that *Candida* spp. biofilms are highly resistant to amphotericin-B and fluconazole, postulating that echinocandins might be more effective [14]. However, several echinocandin-resistant strains have emerged, and high concentrations of these drugs may even facilitate biofilm formation [15].

Given the problems described above, new pharmacological strategies are needed to fight *Candida* spp. infections. One approach is to evaluate the antifungal or antibiofilm effects of “non-antimicrobial” drugs with well-known pharmacological profiles. For instance, prostaglandin synthesis inhibition by non-steroidal anti-inflammatory drugs (NSAIDs) has been shown to decrease biofilm formation and to potentiate the effect of conventional antifungals [16,17]. Prostaglandins are small lipid mediators that participate in physiopathological responses like pain, fever, inflammation, and immune system modulation. In mammalian cells, arachidonic acid from membrane phospholipids is used by cyclooxygenase (COX) isoenzymes and then by prostaglandin synthases to produce specific prostaglandins, including prostaglandin E_2_ (PGE_2_). PGE_2_ is essential for biofilm development and establishment [18,19]. The use of aspirin and other NSAIDs reduces yeast-to-hypha conversion in clinical isolates resistant to fluconazole, in addition to inhibiting the adhesion process [20]. Moreover, various NSAIDs potentiate the effect of fluconazole on *C*. *albicans* isolates that are highly resistant to this antifungal drug [20].

Furthermore, nitric oxide (NO) has been evaluated for use against bacterial and fungal biofilms, as endogenous NO plays an important role in immunity. In the presence of a pathogen, NO acts as a toxic agent towards the infectious organism through complex redox mechanisms involving generation of free radicals and oxidative stress, exerting a static or cidal effect against infectious agents of any class, including fungi [21]. NO donor compounds have been effective in eradicating biofilms of *Staphylococcus aureus* and *Candida albicans* [22,23]. In addition, it has been reported that hyphal cells are more sensitive to NO than planktonic cells [24].

Two novel nitric oxide- releasing aspirins (NO-ASA), NCX-4040 and NCX-4016, have recently been proposed as treatments for various cardiovascular and inflammatory conditions, as these compounds provide both the anti-inflammatory effects of aspirin and the multiple beneficial effects of NO, such as endothelial protection [25]. In this study, we provide the first demonstration of the antibiofilm activity of NO-ASA on fluconazole-resistant *C*. *albicans* isolates, obtained from DS patients, in an *in vitro* model.

## Materials and methods

### Strains

*Candida* spp. isolates from the oral mucosa of patients with DS were provided by Dr. Ximena Lee and Leyla Gómez, Faculty of Dentistry, University of Chile [26]. All isolates were cryopreserved at -20°C in glycerol until use. Fluconazole-resistant *C*. *albicans* ATCC 10231 and susceptible ATCC 90029 were used as the reference strains.

### Disk diffusion susceptibility assay

Thirty DS clinical isolates of *Candida albicans* were grown on Sabouraud chloramphenicol agar plates (Biokar Diagnostics®) and incubated at 37°C for 24 h. Yeast cells from a standardized suspension (0.5 McFarland) were then inoculated using a sterile cotton swab, and drug-containing discs were placed on the center of the agar plate. For susceptibility screening, discs contained only fluconazole (25 μg, OXOID®). Inhibition zone diameters were measured after incubating the plates for 24 h at 37°C. Strains with an inhibition zone diameter ≤14 mm were considered resistant to fluconazole as per Clinical Laboratory Standards Institute (CLSI) breakpoints [27]. According to these standards, we identified 6 fluconazole-resistant strains, used in the remaining portions of the study: 17p, 18r, 29p, 35r, 42p and 50r. For combination studies, commercial sterile blank discs (OXOID^®^) were impregnated with 25 μg fluconazole (Sigma Aldrich) in combination with 25 μg aspirin (Sigma Aldrich) or NO-releasing aspirin (NCX-4040, Santa Cruz Technologies). Blank discs containing each drug alone were used as controls, as well as discs containing only vehicle (DMSO, Sigma Aldrich). Standard strains ATCC 10231 and 90029 were used as resistant and susceptible strains, respectively.

### Induction of morphogenesis

A standardized suspension (0.5 McFarland) obtained from planktonic cultures was incubated in RPMI-1640 medium (Sigma Aldrich) supplemented with FBS (10% v/v) (Gibco) and grown for 12 to 18 h at 28°C. Cells were then centrifuged at 6000 rpm, resuspended, and counted (Neubauer Chamber) to obtain a concentration of 1x10^6^ cells/mL. To induce hyphae formation, cells were incubated at 37°C for 3 h in the presence or absence of the drugs at final concentrations of 835 μM for fluconazole and 125 μM or 500 μM for aspirin or NO-ASA. After incubation, a light microscope was used to identify hyphal cells, and the percentage of hyphal cells was calculated as the number of hyphal cells divided by the total number of cells. At least 10 microscopic fields (approximately 200 cells) were observed under the microscope (40x magnification). Cells were categorized as budded or non-filamentous (less than three joined cells), or b) filamentous cells including true hyphae (no constriction at the septa, walls remaining parallel throughout the hyphae, and branching perpendicular to the cell walls), or pseudohyphal. Several positions per slide were examined to ensure a representative selection of cells.

### Adhesion

One hundred microliters from a standardized cell suspension (0.5 McFarland) were added to each well in a 96-well polystyrene plate in the presence or absence of NO-ASA (125 μM, 500 μM). The control contained fluconazole (800 μM) (Sigma Aldrich). Plates were incubated at 37°C for 4 h without shaking. After incubation, adhered cells at the bottom of the wells were washed three times with 100 μL of PBS. Next, 135 μL of crystal violet (0.1% p/v) were added to each well and incubated for 5 min at RT. Supernatant was discarded, and cells were washed three times with PBS. Finally, 200 μL acetic acid (10% v/v) were added to each well, and the supernatant was read at 590 ηm in a 96-well plate reader (Infinite F50, Tecan®). Results were expressed as percentage of adhesion as compared to the control group with only vehicle (DMSO).

### Biofilm formation and viability assays

100 μL of a standardized suspension (1x10^6^ cells/mL) were added to each well in a 96-well polystyrene plate and incubated for 24 h at 37°C. Supernatant was discarded, and biofilms were washed with sterile PBS to remove non-adherent cells. For viability assays, cells were treated with serial dilutions of fluconazole, ASA, and NO-ASA for 24 h at 37°C. Biofilm viability was quantified using XTT (Life Technologies) reduction assay, as described previously [28]. IC_50_ values were determined for each drug alone.

### Scanning electron microscopy

Three milliliters of a standardized cell suspension (1x10^6^ cells/mL) were added on a coverslip inside a 10 mL cell culture plate and incubated for 24 h at 37°C. Supernatant was then discarded, and biofilms were washed three times with sterile PBS. Biofilms were incubated with 1mM NO-ASA for 24 h at 37°C in RPMI-1640 medium. Controls were incubated in the absence of the drug. Samples were then fixed with 2.5% glutaraldehyde in 0.1 M sodium cacodylate buffer for 2 h, washed with the same buffer, and post-fixed in osmium tetroxide. Dehydration was achieved by placing samples in a series of ethanol gradients [29,30]. Samples were dried and placed in a critical point dryer (Autosamdri-815 Series A) for 45 min. Once dried, samples were coated with gold using a Denton Vacuum Desk V system. A JEOL JSM IT 300 LV scanning electron microscope was used to observe the biofilms.

### Effect of exogenous PGE_2_ and NO scavenging on biofilm susceptibility to NO-ASA

Formed biofilms were treated with 250 μM or 1 mM of NO-ASA or ASA for 24 h at 37°C, in the presence of 1 ηM or 100 ηM PGE_2_ (Cayman Chemical), and cell viability was determined by XTT reduction assay. To evaluate the role of nitric oxide release in the effect exerted by NO-ASA, 24 h biofilms were pre-incubated with Carboxy-PTIO (Sigma Aldrich) 200 μM for 20 min at 37°C. After the pre-incubation period, NO-ASA 250 μM or 1 mM was added and incubated for 24 h at 37°C. At the end of this period cell viability was determined by XTT reduction assay.

### Combination assays

Combination assays were performed using the checkerboard method in 96-well plates. In brief, two-fold serial dilutions of fluconazole and NO-ASA were prepared. One hundred microliters of each concentration of NO-ASA was combined with 100 μL of each concentration of fluconazole in 96-well flat bottom microtitration plates containing 24-h biofilms. Biofilms were then incubated for an additional 24 h in the presence or absence of the drugs. The initial inoculum consisted of 100 μL of a standardized yeast suspension (1x10^6^ cells/mL). To evaluate the interaction, the fractional combination index (CI) was calculated using CompuSyn® v1.0 software, were CI<1.0 indicates synergy and CI>1.0 indicates antagonism.

### Statistical analysis

Results were expressed as mean ± standard deviation and analyzed using two-way ANOVA (GraphPad Prism 6.0). Results were considered statistically significant for *p*-values <0.05. All experiments were performed in triplicate.

## Results

### Effect of NO-ASA on susceptibility of *C*. *albicans* strains to fluconazole

**Fig 1:**
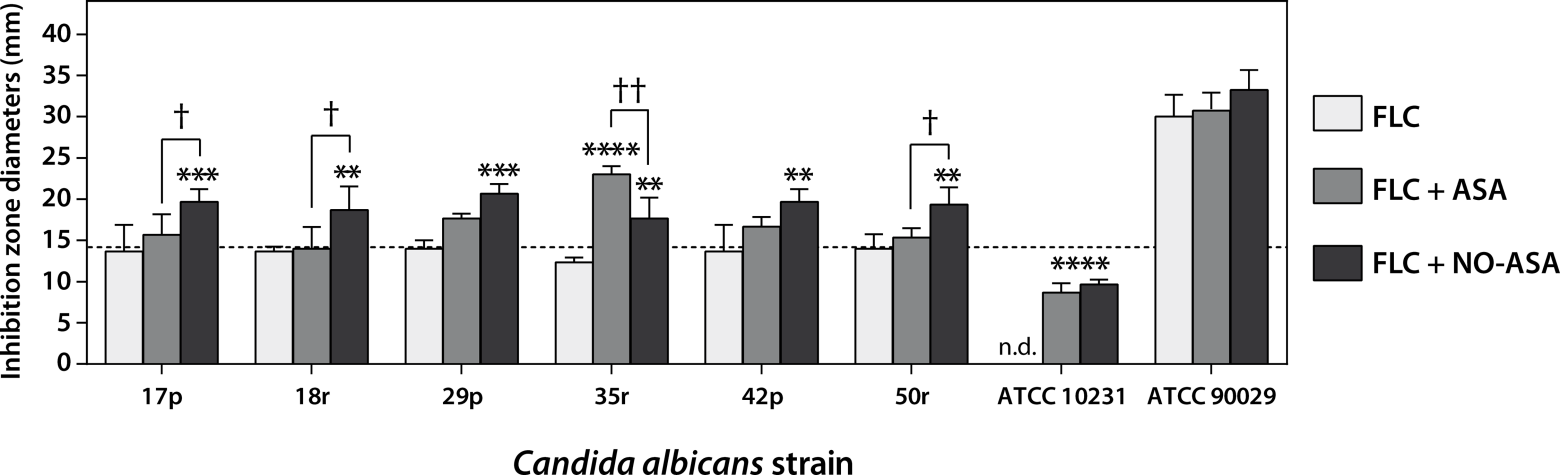
NO-ASA increases the antifungal effect of fluconazole in resistant strains. Bars represent mean ± SD of inhibition zone diameters. Discs contained 25 μg fluconazole, alone or in combination with 25 μg nitric oxide-releasing aspirin or 25 μg ASA. Controls containing only vehicle (DMSO), aspirin (ASA), or NCX-4040 NO-ASA showed no inhibition zone diameters (not shown). *p<0.05; **p<0.01; ***p<0.001; ****p<0.0001 as compared to fluconazole alone; †p<0.05; ††p<0.01 for comparisons between treatments as indicated; nd = inhibition zone not detected (two-way ANOVA).

As shown in Fig 1, nitric oxide-releasing aspirin (NO-ASA) significantly increased the inhibition zone diameter around the fluconazole disc in the six clinical isolates tested: 17p, 18r, 29p, 35r, 42p, and 50r. Notably, NO-ASA increased inhibition zone diameters to values above 14 mm, which is the breakpoint for resistant strains (dashed line). Because other authors have reported a potentiating effect of aspirin on fluconazole, we compared the effects of aspirin and NO-ASA. NO-ASA showed a stronger effect, as aspirin exerted a statistically-significant potentiation only in the 35r strain at the same NO-ASA concentration (25 μg). Although fluconazole alone had no effect on ATCC 10231, combining fluconazole with aspirin or NO-ASA made the strain more susceptible, with detectable inhibition zone diameters. On the other hand, ASA and NO-ASA did not increase the effect of fluconazole on susceptible reference strain ATCC 90029. It is important to note that neither aspirin nor NO-ASA alone exerted any antifungal effect, as discs containing only these drugs did not inhibit growth (data not shown). To corroborate whether NO-ASA lack of antifungal effect is dependent on the culture medium, we performed a microdilution antifungal susceptibility testing following CLSI guidelines in RPMI medium for strains ATCC 10231, ATCC 90029 and 29p. Results indicate that NO-ASA (62 μM to 1 mM) is not able to affect significantly *C*. *albicans* growth (S1 Fig).

### Inhibition of adhesion to abiotic surfaces

**Fig 2:**
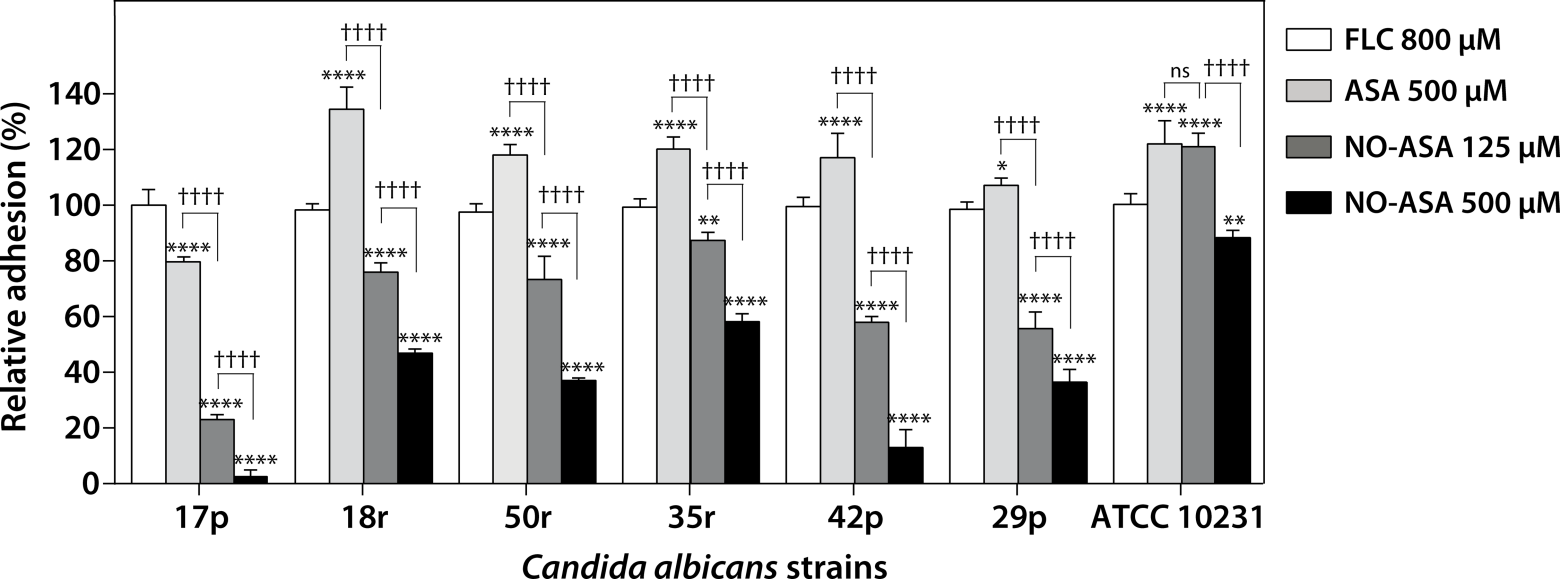
NO-ASA inhibits the adhesion of *C*. *albicans* to abiotic surfaces. Bars represent mean ± SD of percentage of cells adhered to the bottom of wells as compared controls for each strain assayed. Due fluconazole 800 μM did not affect adhesion as compared to untreated strains it was used as control for the analysis. **p<0.01; ****p<0.0001 as compared to fluconazole alone (white bars); ††††p<0.0001 for comparisons between treatments as indicated; ns = no significant difference (two-way ANOVA).

The ability of *C*. *albicans* to adhere to abiotic surfaces is a critical event for biofilm-associated DS. Therefore, we evaluated whether NO-ASA affected the adhesion of the *C*. *albicans* isolates to polystyrene microtiter plates. Fluconazole 800 μM did not affect adhesion of *C*. *albicans* strains assayed when compared with untreated control (data not shown). Fig 2 shows that 125 μM NO-ASA inhibited adhesion in all strains, with reductions in adhesion ranging from 20% in the 35r to 77% in the 17p strain. This effect was dose-dependent, as 500 μM NO-ASA resulted in a stronger inhibition, with reductions ranging from 45% in the 35r to 97% in the 17p strain. It is noteworthy that 500 μM aspirin decreased adhesion only in the most susceptible strain, 17p, and actually increased adhesion for the other strains assayed as compared to fluconazole treated controls. On the other hand, 500 μM NO-ASA decreased adhesion in the ATCC 10231 strain, while aspirin increased adhesion at the same concentration instead.

### NO-ASA affects morphogenesis of *C*. *albicans*

**Fig 3:**
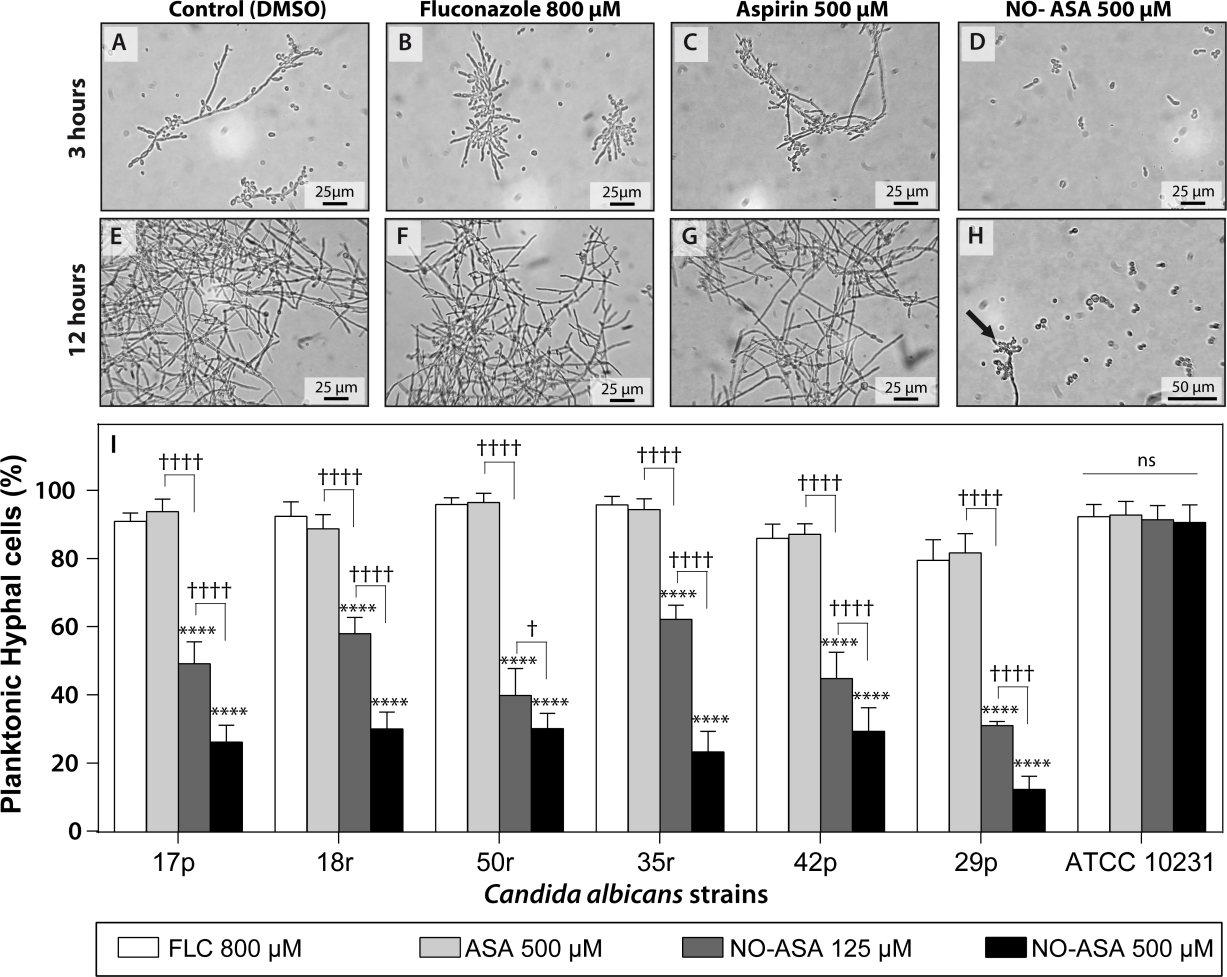
NO-ASA inhibits *C*. *albicans* planktonic morphogenesis. *Upper Panel*: light microscopy photographs showing representative filamentous cells (hyphae or pseudohyphae) for the 17p strain after 3 h or 12 h of incubation with DMSO **(A, E)**; 800 μM fluconazole **(B, F)** or 500 μM aspirin **(C, G).** Cells treated with 500 μM NO-ASA **(D, H)** showed mainly budding yeast cells and scarce filamentous cells (arrow in H). *Lower panel*: Bars represent mean ± SD of percentage of filamentous cells as compared to untreated controls for each strain assayed after 3 hours of incubation (untreated control bars were omitted for clarity). ****p<0.0001 as compared to fluconazole alone (white bars); †p<0.05; †††p<0.0001 for comparisons between treatments as indicated; ns = no significant difference (two-way ANOVA).

One of the main virulence factors of *C*. *albicans* strains is the ability to change from yeast to filamentous cells. Hyphae have been identified as the principal cells responsible for extracellular matrix production in biofilms as well as other resistance mechanisms. We induced yeast-to-hypha conversion in the presence of NO-ASA, aspirin, and fluconazole and evaluated the percentage of filamentous cells as compared to untreated controls (DMSO) for each strain. Fig 3A–3D are representative images for the 17p strain after 3 hours induction of morphogenesis in the absence (DMSO untreated control) or presence of fluconazole, aspirin, or NO-ASA. While 800 μM fluconazole and 500 μM aspirin did not affect the emergence of filamentous cells cells, 500 μM NO-ASA produced an obvious effect, as budding yeast were the predominant cells in the samples even after 12 hours of incubation (Fig 3D and 3H), were true hyphae predominates in all the other treatments. Fig 3 panel I represents quantification of the percentage of filamentous cells after 3 hours of drug treatments for all strains. NO-ASA at both concentrations assayed, 125 μM and 500 μM, decreased the presence of filamentous cells in a concentration-dependent manner in all clinical isolates. On the other hand, none of the treatments affected the morphogenesis of the reference strain ATCC 10231.

**Fig 4:**
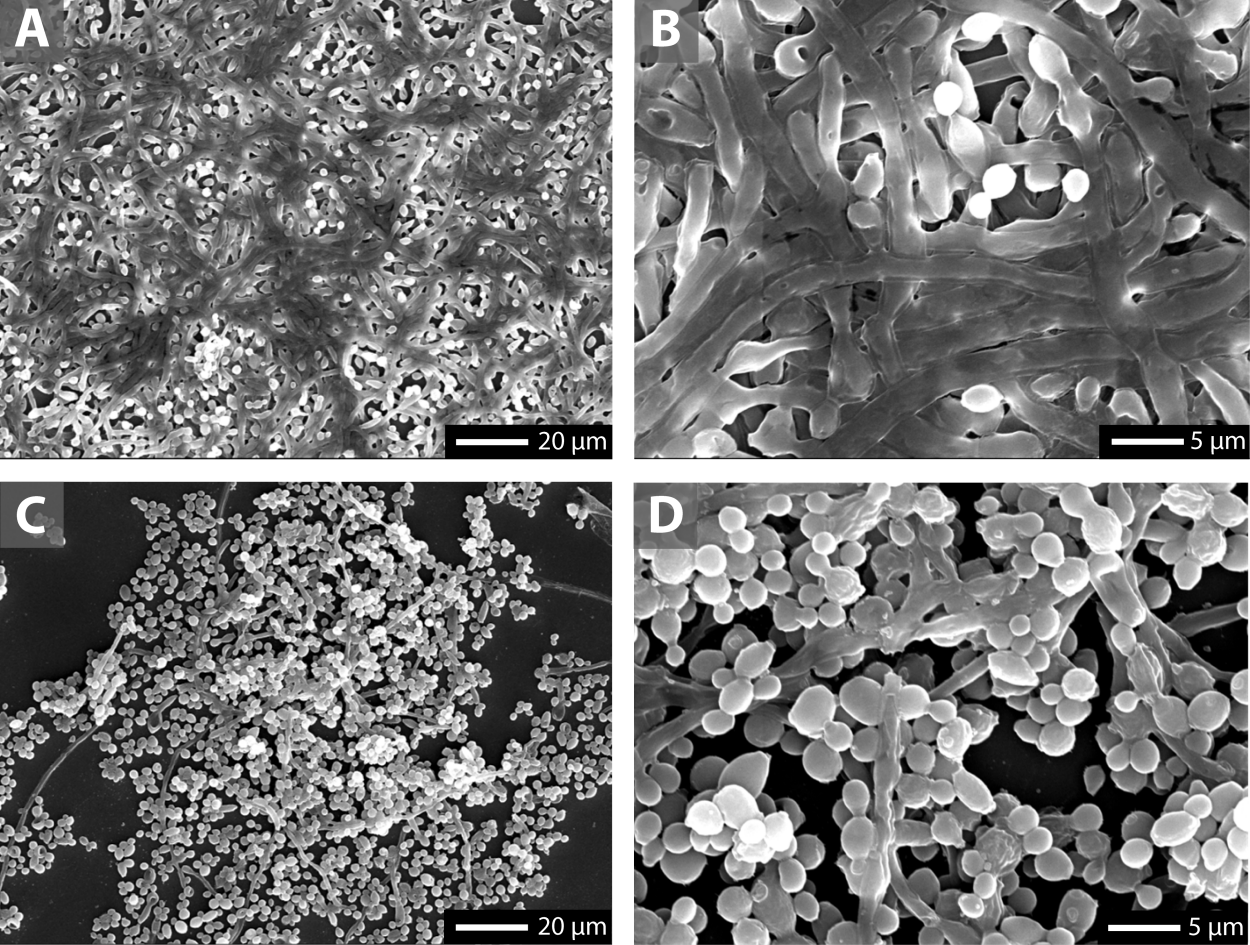
Biofilm microarchitecture is affected by NO-ASA treatment. Representative SEM analysis of 17p strain biofilms on coverslips in the presence or absence of 1 mM NO-ASA. Control biofilms were composed of a dense layer of filamentous cells **(A, B)**. Treatment with NO-ASA reduced the density of the biofilm layers and decreased the presence of filamentous cells **(C, D)**.

To corroborate these results, we evaluated whether the effects exerted by NO-ASA on planktonic cell morphology might also occur in biofilm-forming cells. We analyzed formed biofilms using scanning electron microscopy (SEM). Fig 4A and 4B shows that in untreated 17p strain cells, biofilms were mainly composed of filamentous cells. On the other hand, biofilms treated with 500 μM NO-ASA showed a significantly decreased presence of hyphal cells (Fig 4C and 4D), consistent with results in planktonic cultures.

### Biofilm viability

Once we established that NO-ASA affects adhesion and morphogenesis, we evaluated the effect of this drug on the viability of 24-h biofilms using XTT reduction assay [28]. Viability was determined after 24-h incubation of biofilms with serial dilutions of NO-ASA to obtain IC_50_ values for each strain assayed (Table 1). Among the clinical isolates, the 29p strain was the most susceptible to the effect of NO-ASA on biofilm viability with an IC_50_ of 300 μM, while the most resistant was 50r (IC_50_ 759 μM). On the other hand, although the ATCC 10213 strain showed only a modest response to NO-ASA in the other assays, its viability was significantly affected by this drug, with an IC_50_ of 273 μM. It is important to note that neither fluconazole nor aspirin had an inhibitory effect on the viability of formed biofilms even at concentrations higher than those tested for NO-ASA, 800 mM and 2 mM, respectively (data not shown).

**Table 1:** IC_50_ values of NO-ASA for *C*. *albicans* biofilms from denture stomatitis isolates.

*C*. *albicans* strain	IC_50_ (μM)
**29p**	300.9±30.1
**17p**	564.9±31.4
**18r**	500.8 ± 28.2
**35r**	354.8±17.6
**42p**	499.7±25.0
**50r**	758.8±15.3
**ATCC 10231**	298.2±40.4

Biofilms were formed for 24 h at 37°C and then incubated for an additional 24 h with serial dilutions of NO-ASA. IC_50_ values were determined using XTT reduction assay (see Methods). Values represent mean ± SD.

### Effect of exogenous PGE2 on NO-ASA antibiofilm activity

**Fig 5:**
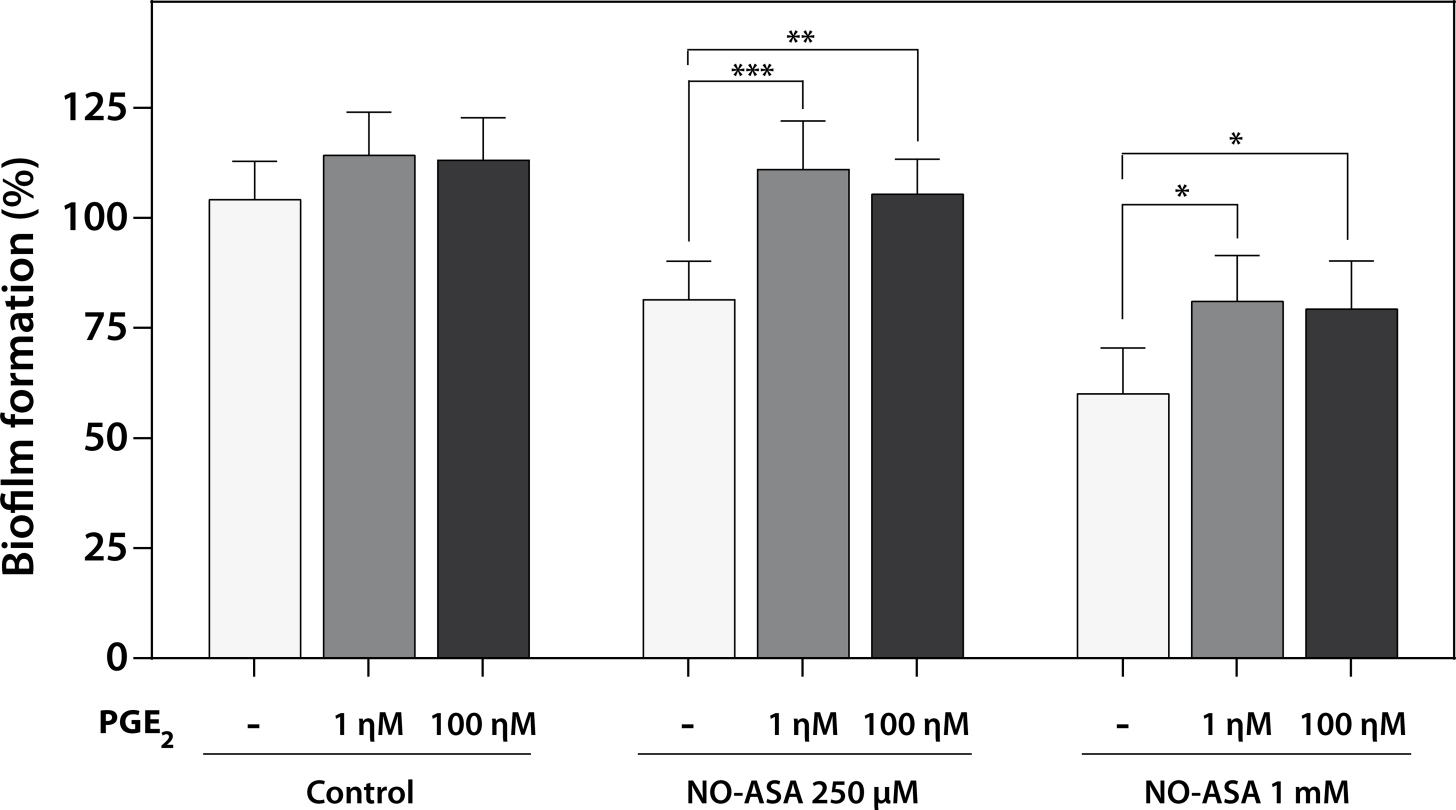
PGE_2_ prevents the antibiofilm effect of NO-ASA. 24-h biofilms were incubated with 250 μM or 1 mM NO-ASA for an additional 24 h in the presence or absence of exogenously-added PGE_2_. Bars represent the mean ± SD biofilm formation quantified through XTT reduction assay. *p<0.05; **p<0.01; ***p<0.001; ****p<0.0001 as compared to NO-ASA-treated biofilms in the absence of PGE_2_.

Because it has been reported that COX inhibitors modulate the process of biofilm formation and viability by inhibiting PGE_2_ synthesis, we wanted to address whether NO-ASA might act also on biofilms via this mechanism. We treated 17p-strain biofilms with 250 μM and 1 mM NO-ASA in the presence of 1 ηM or 100 ηM PGE_2_. Fig 5 shows that the addition of PGE2 to 24 h biofilms did not increase their development. On the other hand, presence of PGE_2_ completely inhibited the effect of 250 μM NO-ASA on viability. For 1 mM NO-ASA, the effect of PGE_2_ was lower but still statistically significant as compared to NO-ASA alone. These results seem to indicate that the effect of NO-ASA could be mediated at least in part by inhibition of PGE_2_ synthesis.

### Role of nitric oxide in the antibiofilm effect of NO-ASA

**Fig 6:**
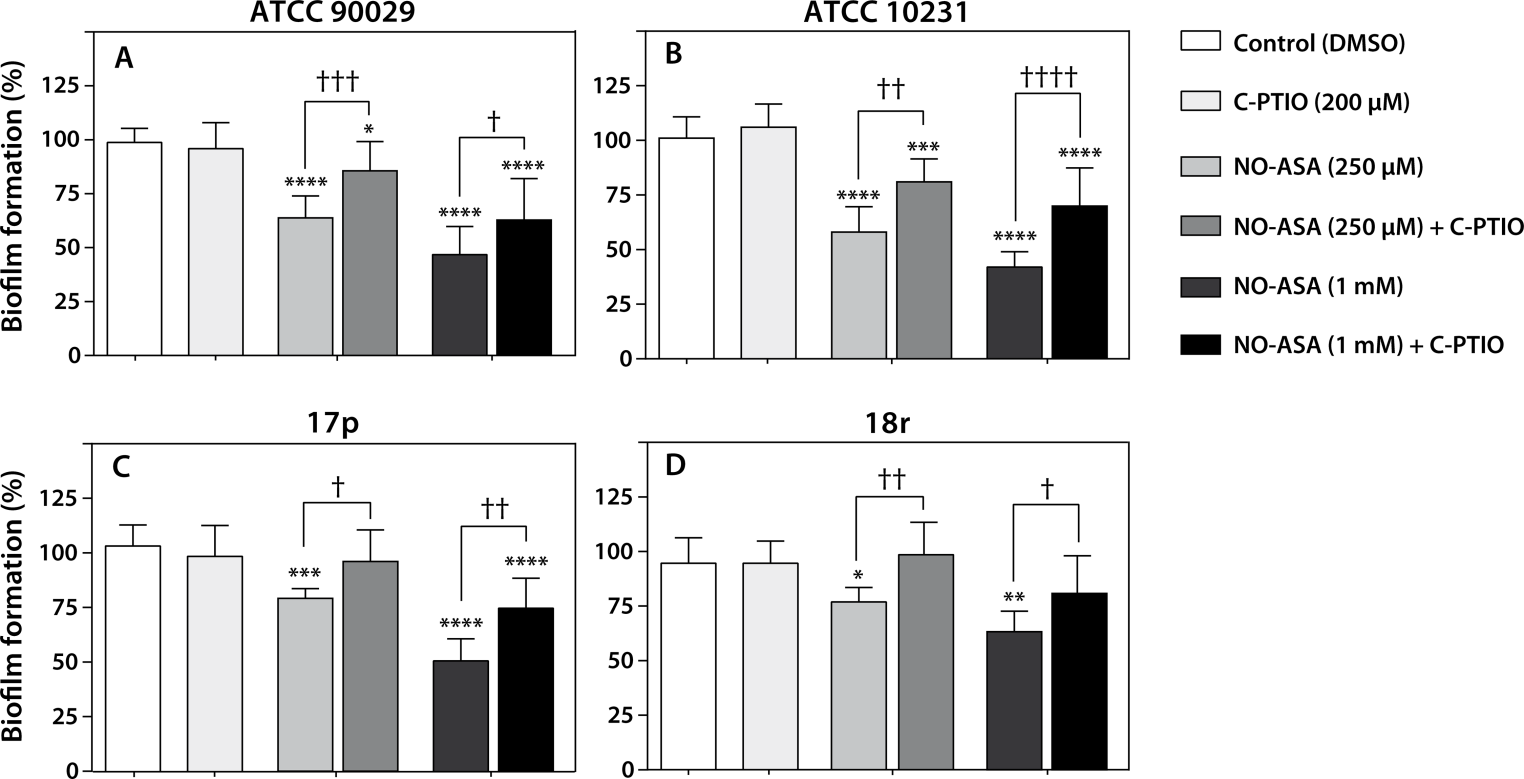
Nitric Oxide scavenger prevents NO-ASA antibiofilm effect. Biofilms were pretreated with 200 μM carboxy-PTIO for 20 min at 37°C. Then NO-ASA 250 μM or 1 mM was added and biofilms were incubated for 24h at 37°C. Bars represent mean ± SD of biofilm formation quantified trough XTT reduction assay. * p<0.05; ** p<0.01; *** p>0.001; ****p>0.0001 as compared with control (DMSO). †p<0.05; ††p<0.01; ††† p>0.001; ††††p>0.0001 as compared between groups as indicated (one way ANOVA).

To corroborate our hypothesis that nitric oxide could be mediating part of the antibiofilm activity seen with NO-ASA, we pretreated 24h biofilms with 200 μM Carboxy-PTIO, an intracellular nitric oxide scavenger, for 20 min at 37°C. After the pre-incubation period, biofilms were treated with 250 μM or 1mM of NO-ASA and cell viability was determined as described above. Fig 6 shows that Carboxy-PTIO prevents partially the antibiofilm effect of 250 μM NO-ASA in a significant manner for ATCC 90029 and ATCC 10231 strains as compared with untreated control. Interestingly, in clinical strains 17p and 18r, the prevention of antibiofilm effect was complete (Fig 6 panels C and D, center bars). However, when cells were treated with 1mM NO-ASA, the effect of Carboxy-PTIO decreases (Fig 6 panels A-D, right bars). These results indicate that nitric oxide is also involved in the antibiofilm effect of NO-ASA.

### Isobolograms

It has been reported that NSAIDs potentiate the effect of fluconazole on biofilms [20]. Therefore, we also evaluated the viability of biofilms treated with combinations of fluconazole and NO-ASA. Based on reports from similar *in vitro* studies regarding effective concentrations of fluconazole against biofilms [20,31], we used fluconazole at serial dilutions ranging from 1000 μg/mL to 15,625 μg/mL. Using the checkerboard method, fluconazole was combined with NO-ASA at concentrations ranging from 1 mM to 15.6 μM. In most of the concentrations assayed, fluconazole increased biofilm viability, whereas the opposite occurred for NO-ASA-treated cells. Therefore, after evaluating the combinations using CompuSyn software, the interaction was defined as “antagonistic” (S1 Table). This could be attributed to the resistant nature of the clinical isolates.

## Discussion

Although DS is the most common form of oral candidiasis, current pharmacological treatments show little long-term efficacy against the disease. In addition, in the last years prevalence of fluconazole resistance has increased. This is of special relevance for denture stomatitis, were in unresponsive cases systemic fluconazole is often used as last-line strategy. The main limitation of classical antifungals is a lack of antibiofilm activity. For this reason, we were interested in the search of newer antibiofilm agents effective on resistant strains to conventional treatments. Previous studies have noted an antibiofilm effect of COX inhibitors such as aspirin in diverse *in vitro* models for *C*. *albicans* [16,17,20]. On the other hand, nitric oxide-releasing compounds have been studied extensively as antimicrobial and antibiofilm drugs for infections of bacterial and fungal origin [23,32,33]. To our knowledge, this is the first report to evaluate a molecule that could attack biofilms through a combination of both mechanisms. Therefore, our first step was to characterize the potential antifungal effect of NO-ASA. Disk diffusion assay showed that while NO-ASA had no effect on fungal growth in agar media, it efficiently increased susceptibility to fluconazole in resistant *C*. *albicans* strains.

Anti-adhesion properties are extremely important for new antibiofilm molecules, since it is well recognized that the adhesion process is directly associated with the ability of *C*. *albicans* to form biofilms [34]. In DS, adhesion of *C*. *albicans* to acrylic dentures is uncontrolled, due to poor compliance with hygiene measures as well as the inability of conventional topical and systemic antifungals to prevent adhesion of fungi. In our model, NO-ASA significantly affected adhesion (mean reduction of 63%) for all DS strains assayed.

Morphogenesis is also a crucial step in biofilm formation, since yeast-to-hypha conversion is associated with expression of several virulence factors, including adhesins hyphal wall protein 1 (Hwp1), Als1p (agglutinin-like sequence 1 protein), and Als3p, which allow *C*. *albicans* to adhere to oral mucosa and abiotic surfaces such as acrylic denture or tissue conditioners used in prosthodontics [35]. Our results show that NO-ASA not only prevented morphogenesis in planktonic yeasts but also inhibited hyphal development in formed biofilms on abiotic surfaces, according to SEM analysis. This finding is of special relevance, as it suggests that NO-ASA can revert filamentous cells to a less virulent phenotype. Other authors have shown that the clinical isolates that are capable of forming germ tubes are associated with more severe types of DS [36]. It has been reported that aspirin and other COX inhibitors affect hyphal development [16,20]. Similar to the results reported by Alem and Douglas (2004) [16], aspirin was ineffective at 500 μM under our experimental conditions. This result stands in contrast to the findings of Abdelmegeed and Shaaban (2013) [20], who reported that 1 mM and 10 mM aspirin decreased germ tube formation by at least 50%. This result could be explained in part by the fact that we used a lower concentration of aspirin to compare its potency with NO-ASA. However, the fact that NO-ASA was effective at much a lower concentration than the aspirin concentration used by Abdelmegeed and Shaaban (2013) [20] suggests an additional inhibitory effect due to the release of NO. Supporting this concept, Heilman et al. (2013) [24] showed that NO can greatly affect hyphal formation in *C*. *albicans*.

Contrary to previous reports based on XTT reduction assays in the presence of COX inhibitors [16,18,20], aspirin did not affect the viability of biofilms in our model, even at a concentration of 1 mM. This result could be explained in part by differences between our strains and those used in other studies. Previous reports have assayed biofilms with strains isolated from skin lesions [18], DS [16], sputum, the ear, and blood [20], as well as standard ATCC strains [37]. Given that the phenotype of *C*. *albicans* clinical isolates varies depending on environmental stress factors, results using the DS strains used in this study will not necessarily be consistent with the effects observed by others. For example, in a recent study, aspirin was shown to decrease the biofilm viability of clinical invasive candidiasis strains [38]. However, it is important to note that in our model, NO-ASA decreased viability in a concentration-dependent manner in the micromolar range, with a mean IC_50_ of ~300 μM. Moreover, the IC_50_ values obtained are in the same range as plasma concentrations in animals treated with NO-ASA for cardiovascular benefits [39]. Given that other authors have shown that COX inhibitors synergistically potentiate some antifungals [17,38], we used a checkerboard assay to test combinations of fluconazole and NO-ASA. However, in the range of concentrations tested, we obtained antagonistic interactions between the two drugs. This result is associated with the finding that, in our experimental conditions, fluconazole had no effect or even increased biofilm viability, opposing the antibiofilm effect of NO-ASA. It has been reported that other antifungals belonging to echinocandins family also increase biofilm formation at certain range of doses [15]. So, for combination assays this aspect has to be considered.

Initial insights into the role of PGE_2_ synthesis in *C*. *albicans* showed that this eicosanoid seems to be relevant for both formation and maintenance of biofilms [40]; hence, COX inhibitors were a logical approach for inhibiting biofilm formation. Therefore, we wanted to evaluate whether the effect of NO-ASA could be explained in part by inhibition of PGE_2_ synthesis. Although when we added PGE_2_ exogenously biofilms did not increased their development as compared to control, the effect of 250 μM of NO-ASA on biofilm viability was completely reverted by PGE_2_ and partially reverted at high NO-ASA concentrations (1 mM). This result indicates that PGE_2_ can overcome the effect of NO-ASA, as previously described for aspirin [16]. Although this finding seems to indicate a minor role for NO release, aspirin was much less effective or not effective at all as an antibiofilm agent at the same concentrations in all our assays, indicating that adding a NO-releasing group significantly increased the effect of the parent compound. This is corroborated by the prevention of the NO-ASA antibiofilm effect with carboxy-PTIO, a well-known intracellular nitric oxide scavenger.

## Conclusion

In conclusion, nitric oxide-releasing aspirin (NO-ASA, NCX-4040) is an effective antibiofilm agent, with a stronger effect than ASA on fluconazole-resistant *Candida albicans* strains obtained from DS patients. However, there was no synergistic interaction when NO-ASA was combined with fluconazole. Further studies are needed to elucidate the potential of the NO-releasing group as an additional antibiofilm mechanism.

## Supporting information

S1 Fig NO-ASA did not affect planktonic *Candida albicans* growth. Standardized planktonic cultures were obtained and microdilution antifungal susceptibility testing was performed following CLSI guidelines in RPMI medium for strains ATCC 10231, ATCC 90029 and 29p. Results indicate that NO-ASA (62 μM to 1 mM) is not able to affect significantly *C*. *albicans* growth. The values represent mean ± SD of at least three independent experiments.
(EPS)

S1 Table Fluconazole is antagonistic for NO-ASA antibiofilm effect. Combinatory index for fluconazole and NO-ASA combination. 96 well plates were seeded with different strains and treated with serial dilutions of drugs by checkerboard assay according to described in Methods. Data was analyzed using CompuSyn® Software v1.0. The data was entered to the software only when effect of fluconazole was a positive value as is indicated in Results section and Discussion. CI > 1 indicates antagonistic interaction. These values are only for reference since the lack of valid points in fluconazole treatment is inadequate to a correct calculation of parameters.
(DOCX)
