# Supplementary material for: Characterization of a novel antibiofilm effect of nitric oxide-releasing aspirin (NCX-4040) on Candida albicans isolates from denture stomatitis patients
Source: PLoS One. 2017 May 11;12(5):e0176755. doi: 10.1371/journal.pone.0176755 (PMC5426659; doi:10.1371/journal.pone.0176755)
Supplement: S1 Table — Combinatory index for fluconazole and NO-ASA combination. 96 well plates were seeded with different strains and treated with serial dilutions of drugs by checkerboard assay according to described in Methods. Data was analyzed using CompuSyn® Software v1.0. The data was entered to the software only when effect of fluconazole was a positive value as is indicated in Results section and Discussion. CI > 1 indicates antagonistic interaction. These values are only for reference since the lack of valid points in fluconazole treatment is inadequate to a correct calculation of parameters. (DOCX) [file pone.0176755.s002.docx]

**17p strain**

| **Effect of each drug alone** | | | |
| --- | --- | --- | --- |
| **Fluconazole dose (mg/L)** | **Effect** | **NO-ASA dose (µM)** | **Effect** |
| 16,125 | 0,087 | 16,125 | 0.002 |
| 31,25 | 0,067 | 31,25 | 0.061 |
| 62,5 | 0,018 | 62.5 | 0.042 |
|  |  | 125 | 0.219 |
|  |  | 500 | 0.498 |
| **Combinations of drugs** | | | |
| **Fluconazole dose (mg/L)** | **NO-ASA dose (µM)** | **Effect** | **CI** |
| 16,125 | 250.0 | 0.085 | **3.73398** |
| 31.25 | 250.0 | 0.066 | **4.72956** |
| 62.5 | 250.0 | 0.069 | **6.08709** |
| 125.0 | 250.0 | 0.054 | **8.32828** |
| 250.0 | 250.0 | 0.068 | **14.1868** |
| 16.125 | 500.0 | 0.369 | **5.71067** |
| 31.25 | 500.0 | 0.229 | **7.08988** |
| 62.25 | 500.0 | 0.243 | **11.9518** |
| 125.0 | 500.0 | 0.191 | **17.5185** |
| 250.0 | 500.0 | 0.246 | **39.9493** |
| 500.0 | 500.0 | 0.234 | **73.4143** |

**29p strain**

| **Effect of each drug alone** | | | |
| --- | --- | --- | --- |
| **Fluconazole dose (mg/L)** | **Effect** | **NO-ASA dose (µM)** | **Effect** |
| 500.0 | 0.01 | 16.125 | 0.082 |
| 1000.0 | 0.081 | 31.25 | 0.062 |
|  |  | 62.5 | 0.173 |
|  |  | 125.0 | 0.237 |
|  |  | 250.0 | 0.469 |
|  |  | 500.0 | 0.534 |
| **Combinations of drugs** | | | |
| **Fluconazole dose (mg/L)** | **NO-ASA dose (µM)** | **Effect** | **CI** |
| 250.0 | 16.0 | 0.254 | **2.16638** |
| 250.0 | 31.25 | 0.189 | **3.35670** |
| 250.0 | 62.5 | 0.175 | **3.76177** |
| 250.0 | 125.0 | 0.148 | **4.77424** |
| 250.0 | 250.0 | 0.12 | **6.39060** |
| 250.0 | 500.0 | 0.049 | **19.4928** |
| 250.0 | 1000.0 | 0.112 | **7.64431** |

**ATCC 10231 strain**

| **Effect of each drug alone** | | | |
| --- | --- | --- | --- |
| **Fluconazole dose (mg/L)** | **Effect** | **NO-ASA dose (µM)** | **Effect** |
| 16.0 | 0.026 | 16.0 | 0.024 |
| 31.0 | 0.340 | 31.0 | 0.031 |
| 63.0 | 0.013 | 63.0 | 0.083 |
| 125.0 | 0.044 | 125.0 | 0.102 |
| 250.0 | 0.100 | 125.0 | 0.287 |
| 500.0 | 0.161 | 500.0 | 0.387 |
|  |  | 1000.0 | 0.56 |
| **Combinations of drugs** | | | |
| **Fluconazole dose (mg/L)** | **NO-ASA dose (µM)** | **Effect** | **CI** |
| 16.0 | 16.0 | 0.024 | **13.1908** |
| 16.0 | 31.0 | 0.01 | **359.950** |
| 16.0 | 125.0 | 0.099 | **1.64468** |
| 16.0 | 250.0 | 0.274 | **0.92215** |
| 16.0 | 500.0 | 0.32 | **1.47547** |
| 16.0 | 1000.0 | 0.417 | **1.93337** |
| 31.0 | 125.0 | 0.094 | **1.79997** |
| 31.0 | 250.0 | 0.237 | **1.12370** |
| 31.0 | 500.0 | 0.32 | **1.47563** |
| 31.0 | 1000.0 | 0.38 | **2.25975** |
| 63.0 | 125.0 | 0.086 | **2.17455** |
| 63.0 | 250.0 | 0.189 | **1.50950** |
| 63.0 | 500.0 | 0.306 | **1.57643** |
| 63.0 | 1000.0 | 0.392 | **2.14720** |
| 125.0 | 125.0 | 0.072 | **3.51478** |
| 125.0 | 250.0 | 0.208 | **1.34157** |
| 125.0 | 500.0 | 0.366 | **1.20084** |
| 125.0 | 1000.0 | 0.402 | **2.05866** |
| 250.0 | 125.0 | 0.145 | **1.16205** |
| 250.0 | 250.0 | 0.248 | **1.06660** |
| 250.0 | 500.0 | 0.322 | **1.46429** |
| 250.0 | 1000.0 | 0.372 | **2.33991** |
| 500.0 | 125.0 | 0.146 | **1.27435** |
| 500.0 | 250.0 | 0.271 | **0.94911** |
| 500.0 | 500.0 | 0.369 | **1.18705** |
| 500.0 | 1000.0 | 0.429 | **1.84060** |

**Supplementary Table 1. Fluconazole is antagonistic for NO-ASA antibiofilm effect. Combinatory index for fluconazole and NO-ASA combination.** 96 well plates were seeded with different strains and treated with serial dilutions of drugs by checkerboard assay according to described in Methods. Data was analyzed using CompuSyn® Software v1.0. The data was entered to the software only when effect of fluconazole was a positive value as is indicated in Results section and Discussion. CI > 1 indicates antagonistic interaction. These values are only for reference since the lack of valid points in fluconazole treatment is inadequate to a correct calculation of parameters.
